# Methicillin resistant staphylococci isolated in clinical samples: a 3-year retrospective study analysis

**DOI:** 10.2144/fsoa-2020-0183

**Published:** 2021-02-04

**Authors:** Abdel Karim Foloum, Luria Leslie Founou, Sabrina Karang, Yolande Maled, Cedrice Tsayem, Martin Kuete, Michel Noubom, Raspail Carrel Founou

**Affiliations:** 1 Department of Biological Sciences, Higher Institute of Medical Technology, Yaoundé, Cameroon; 2 Department of Food Safety & Environmental Microbiology, Centre of Expertise & Biological Diagnostic of Cameroon (CEDBCAM), Yaoundé, Cameroon; 3 AMR Insights, Ambassador Network; 4 Bioinformatics & Applied Machine Learning Research Unit, EDEN Foundation, Yaoundé, Cameroon; 5 Department of Clinical Biochemistry, Centre of Expertise & Biological Diagnostic of Cameroon (CEDBCAM), Yaoundé, Cameroon; 6 Department of Reproductive & Child Health, Centre of Expertise & Biological Diagnostic of Cameroon (CEDBCAM), Yaoundé, Cameroon; 7 Department of Biological Sciences, Faculty of Medicine & Pharmaceutical Sciences, University of Dschang, Dschang, Cameroon; 8 District Hospital of Dschang, Dschang, Cameroon

**Keywords:** antimicrobial resistance, Cameroon, clinical samples, developing countries, methicillin resistant staphylococci, MRSA, multi-drug resistance, sub-Saharan Africa

## Abstract

**Aim:**

To determine the prevalence and describe the antimicrobial resistance patterns of circulating methicillin-resistant staphylococci (MRS) isolated from clinical specimens during a 3-year period in Yaoundé, Cameroon.

**Materials & methods:**

From January 2017 to December 2019, 1683 clinical samples were plated onto Mannitol salt agar. Bacterial identification was performed followed by antibiotic susceptibility testing. Data were analyzed using R program.

**Results:**

Staphylococci were identified in 90 (5.35%) of the 1683 clinical samples. Among these, 83.33% were MRS with 78.67% being methicillin-resistant *Staphylococcus aureus* (MRSA). The prevalence of MRS infection increased significantly with age.

**Conclusion:**

The study offers a good baseline for surveillance intervention to contain antimicrobial resistance and highlights the need to strengthen antimicrobial stewardship and infection, prevention and control programs in the country.

## Lay abstract

Antimicrobial resistance is an increasing concern worldwide with several health, societal and economic consequences. Methicillin-resistant staphylococci (MRS) is among the leading cause of difficult-to-treat infections. There is a lack of data regarding the burden of MRS in the private healthcare sector in Cameroon. Hence, the current study was performed to fill the observed gaps. We found that MRS is highly prevalent (80%) in the country and increased significantly with age. Our study provides evidence for reinforcing surveillance and prevention measures to contain MRS and by extension antimicrobial resistance in Cameroon.

## Graphical abstract

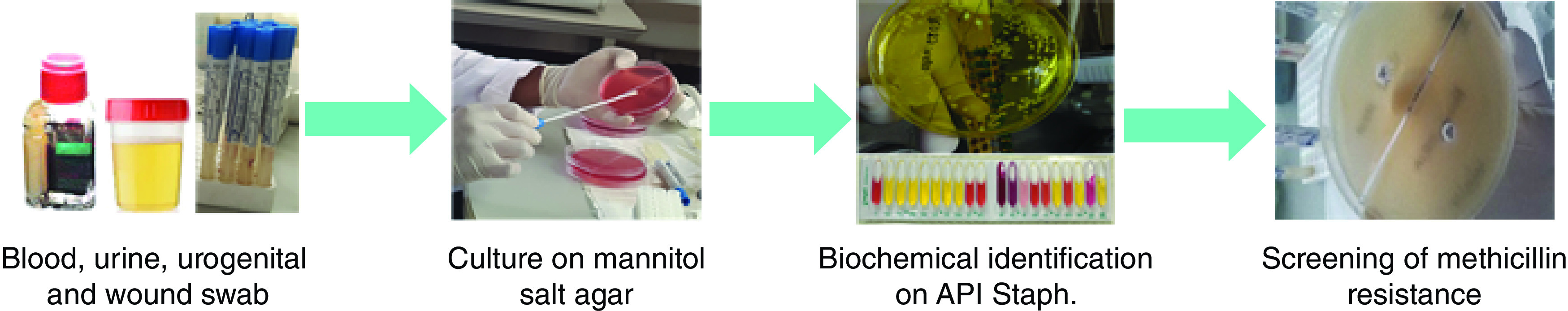

*Staphylococcaceae* is a ubiquitous family of Gram-positive cocci that are natural inhabitants of skin, mucosa and anterior nares. It is grouped into coagulase-negative (CoNS) and -positive (CoPS) staphylococci based on their ability to produce the coagulase enzyme [1]. Although staphylococcal species are important commensal colonizers of animals and humans, they have been involved in a variety of pathologies including pimples, abscesses, septicemia, meningitis, pneumonia and toxicosis [2]. Staphylococci are a major cause of infections in hospital and community settings [1] and the emergence of methicillin-resistant staphylococci (MRS) including methicillin-resistant *Staphylococcus aureus* (MRSA) and methicillin-resistant coagulase-negative staphylococci (MR-CoNS) highlights their public health significance worldwide [3].

Several studies reported high prevalence of MRSA isolated in clinical samples with 80.4, 56.2 and 37.43% in Cameroon, Afghanistan and Ethiopia, respectively [3,4]. Moreover, the principal sources of MRSA infections were pus, blood, ear discharge, nasal and throat swab [2,3]. MRSA and methicillin-resistant coagulase-negative staphylococci (MR-CNS) are recognized for their ability to exhibit multi-drug resistance (MDR), the concomitant resistance to three or more antibiotic families, that leads to increasing treatment failure in clinical practice [4,5] and thereby, to high fatality rates [2,4].

The increasing prevalence of MRS thus poses a serious public health threat worldwide and remain a major challenge in the management of infectious diseases in humans and animals [1]. However, due to the lack of studies regarding this threat, there is a dearth of information about MRS in clinical samples in Cameroon. The aim of this study was to describe and analyses the trends of antimicrobial resistance profiles of circulating MRS strains among community and hospitalized patients in private healthcare sectors in Yaoundé, Cameroon.

## Materials & methods

### Study design & area

Yaounde, the political capital of Cameroon, is an urbanized area of 183 km^2^ populated by 410,000 inhabitants in 2019. The department of Mfoundi, Chief town of the Centre Region is home of the most important Cameroonian institutions. The present work was a laboratory-based retrospective study conducted over a 3-year period from January 2017 to December 2019. In this study, patients attending private healthcare sector including four medical health centers, which provide various services (internal medicine, obstetrics and gynecology, pediatrics and child health and general medicine) and one laboratory located in the Mfoundi, were included in the study. The study was carried out as per the STROBE statement checklist (https://www.strobe-statement.org/?id=available-checklists).

### Laboratory analysis

#### Bacterial isolation & identification

Selected specimens including urine, uro-genital swabs, wound swabs and blood, were analyzed within the premises the Department of Clinical Microbiology of the Center of Expertise and Biological Diagnostic of Cameroon (CEDBCAM). Additionally, specimens were collected from both asymptomatic and clinically ill patients, hence, it was not possible to distinguish infection from colonization. Additional information on participants including age, gender, previous antibiotic intake and type and associated medical conditions was also collected. All specimens were cultured to Mannitol salt agar and incubated for 18–24 h at 37°C. Each morphotype colony growing on mannitol salt agar were subjected to Gram staining, catalase, oxidase, coagulase and DNase tests, followed by biochemical identification with API Staph (BioMérieux, Marcy l’Etoile, France) according to the manufacturer’s instructions. Pure colonies were stored into Tryptone Soya Broth supplemented with 30% glycerol at -20°C for the future analysis.

#### Phenotypic MRSA screening & antimicrobial susceptibility testing

Each unique morphotype underwent a two-step phenotypic screening for methicillin resistance using ROSCO DIAGNOSTICA for MRSA screening (Taastrup, Denmark) and the cefoxitin disc test as per the manufacturer’s instructions and the Clinical Laboratory and Standards Institute (CLSI, 2016) guidelines, respectively. Antibiotic susceptibility testing was performed using the Kirby–Bauer disc diffusion method [6]. Bacterial suspensions were prepared sterile saline solution by peaking up 2–4 colonies from pure culture and adjusting it to a 0.5 MacFarland solution. Penicillin, cefoxitin, amikacin, gentamicin, netilmicin, ciprofloxacin, levofloxacin, doxycyclin, tetracyclin, nitrofurantoin, clindamycin, erythromycin, azithromycin, fosfomycin, fusidic acid, vancomycin and trimethoprim-sulfamethoxazole were the antibiotics being tested. The resulting breakpoints were interpreted according to Clinical Laboratory and Standards Institute guidelines [6].

### Data collection & statistical analysis

Information regarding patient personal details, specimen collection, sampling and laboratory results is deposited on the CEDBCAM (www.cedbcam.com) laboratory database under a software named ToolPro Manager for Health. The access to and use of this database is password-protected and restricted to laboratory staff working within the CEDBCAM. Therefore, data collection was undertaken within the CEDBCAM with extraction of isolate information being sourced from this computerized laboratory database. Data received was deduplicated and all the patient information was codified to ensure the ethical confidentially at all times. Participant demographics information including age, gender, isolate information, specimen type and antimicrobial susceptibility testing results were extracted and tabulated in an MS Excel Spreadsheet (version 2016). Upon data extraction, trends in the total number of staphylococcal isolates and their antimicrobial resistance patterns were assessed. These trends were subsequently compared over a 3-year period. R and inZight^®^ (version 3.5.3) were used for the statistical analyses. Discrete variables were expressed as percentages and proportions with trends or associations being assessed using the standard Pearson’s Chi-square (χ^2^) test. A p-value below 0.05 was considered statistically significant.

## Results

### Participant characteristics

Out of the 1683 clinical samples of interest being analyzed over a 3-year period, uro-genital swabs were the most common (67.74%, 1140/1683), followed by urine (25.07%, 422/1683), blood (5.88%, 99/1683) and wound swab (1.31%, 22/1683). The number of specimens increased over the years ranging from 177 in 2017 to 1077 clinical samples in 2019, with females (1332/1683) more frequently attending laboratory than males (351/1683). The age of participants ranged from 1 month to 84 years, with 33 and 31 years being the mean and median age, respectively.

### Epidemiological background of Staphylococci & MRS

Among 1683 clinical samples (blood, urine, uro-genital and wound swabs) analyzed, a 5.35% (90/1683) prevalence of staphylococci was recorded over the 3-year period. This staphylococci prevalence varied across year with a 1.69% (3/177) prevalence in 2017, 8.86% (38/429) in 2018 and 4.55% (49/1077) prevalence in 2019. Among these, over the 3-year period, a total of 83.33% (75/90) were MRS and 16.67% (15/90) were methicillin-susceptible staphylococci with high statistical significance (p = 0.0004). The prevalence of MRS increased across year with 1.33, 46.67 and 52% of MRS being detected in 2017, 2018 and 2019, respectively, with statistical significance (Table 1). Within the MRS, 78.67% (59/75) were MRSA and 21.33% (16/75) were methicillin resistant coagulase negative staphylococci (MR-CoNS). Although MR-CoNS was more prevalent than MRSA in 2017, the MRSA prevalence increased from 45.76 to 54.24% in 2018 and 2019, respectively (Table 1).

**Table 1.** Epidemiological background of methicillin resistant staphylococci, in relation to individual and year-related characteristics.

Variables	MRS			MRSA			MR-CoNS		
	n	Prevalence (%)	p-value	n	Prevalence (%)	p-value	n	Prevalence (%)	p-value
Overall	75	83.33	**0.0004**	59	78.67	-	16	21.33	-
Gender									
Female	52	69.3	0.092	40	67.797	0.579	12	75	0.579
Male	23	30.7		19	32.203		4	25	
Age (years)									
<5	1	1.33	**0.005**	0	0	0.205	1	6.250	0.205
[6–15]	2	2.667		2	3.397		0	0	
[16–25]	10	13.33		9	15.084		1	6.250	
[26–35]	34	45.33		24	40.678		10	62.50	
[36–45]	12	16.00		10	16.949		2	12.50	
>45	16	21.33		14	23.729		2	12.50	
Specimen									
Urine	18	24	0.083	10	16.949	0.052	8	50	0.052
Uro-genital secretion	47	62.66		40	67.797		7	43.75	
Wound	9	12		8	13.55		1	6.250	
Blood	1	1.33		1	1.695		0	0	
Year									
2017	1	1.33	**0.018**	0	0	0.135	1	6.25	0.135
2018	35	46.67		27	45.763		8	50	
2019	39	52		32	54.24		7	43.75	

MR-CoNS: Methicillin-resistant coagulase-negative staphylococci; MRS: Methicillin-resistant staphylococci; MRSA: Methicillin-resistant *Staphylococcus aureus*.

Bold p-values indicate statistical significance.

**Figure 1.**
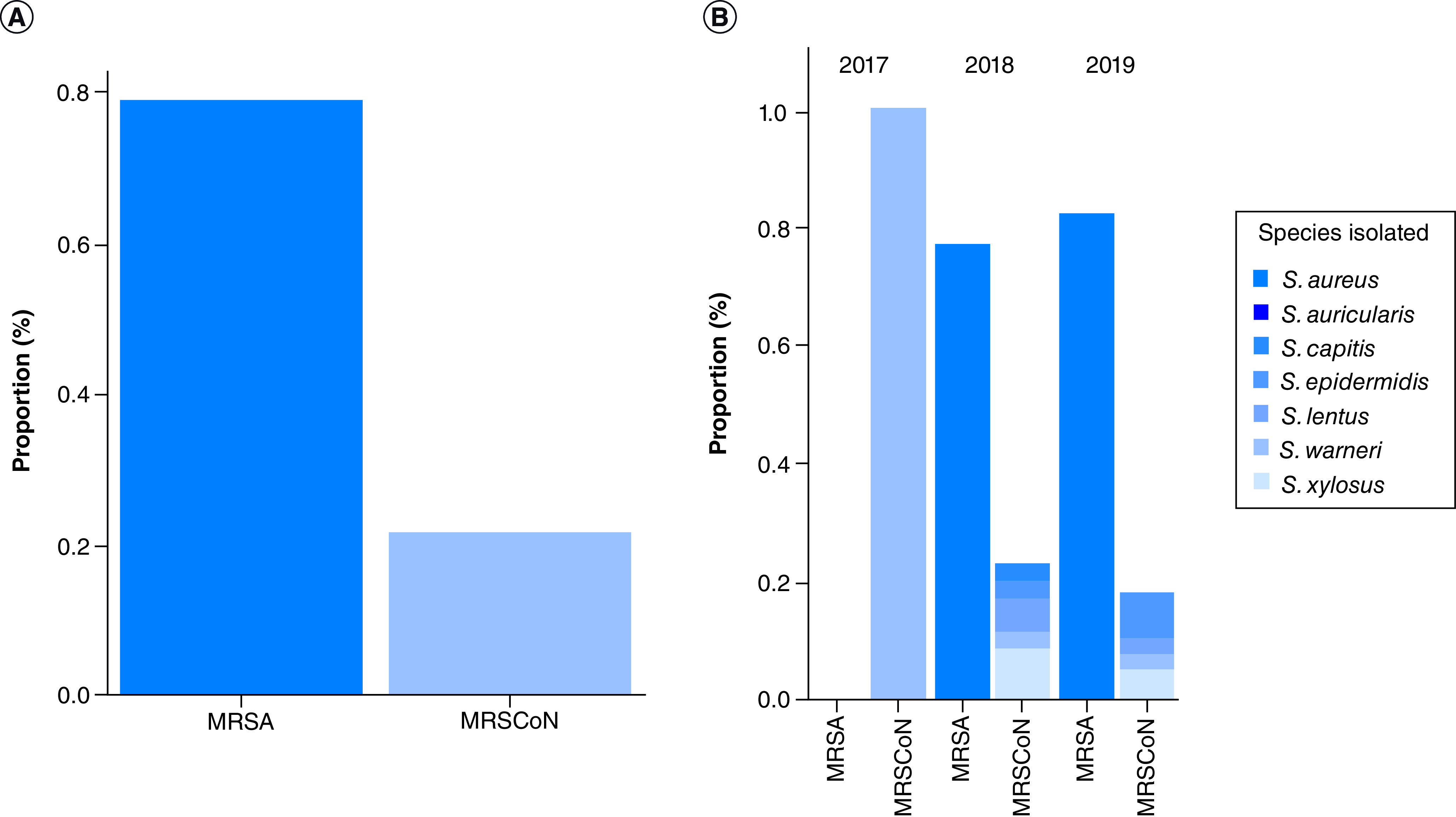
Overall distribution of methicillin-resistant staphylococci (A) and distribution of methicillin-resistant staphylococci species per year (B). MR-CoNS: Methicillin-resistant coagulase-negative staphylococci; MRS: Methicillin-resistant staphylococci; MRSA: Methicillin-resistant *Staphylococcus aureus*.

Overall, *S. aureus* (75.28%) was the most common MRS detected in clinical samples with 75.68% (28/37) and 79.59% (39/49) in 2018 and 2019, respectively (Figure 1). It was followed by *S. xylosus* (8.99%), *S. epidermidis* (4.49%) and *S. lentus* (4.49%). Altogether, females were more positive to MRS than males although it was not statistically significant (69.3 vs 30.7%; p = 0.092). Similarly, when analyzed at the species level, females were more infected by MRSA than males (67.8 vs 32.20%; X^2^ = 0.307; p = 0.579) although no statistical significance was observed (Table 1).

**Figure 2.**
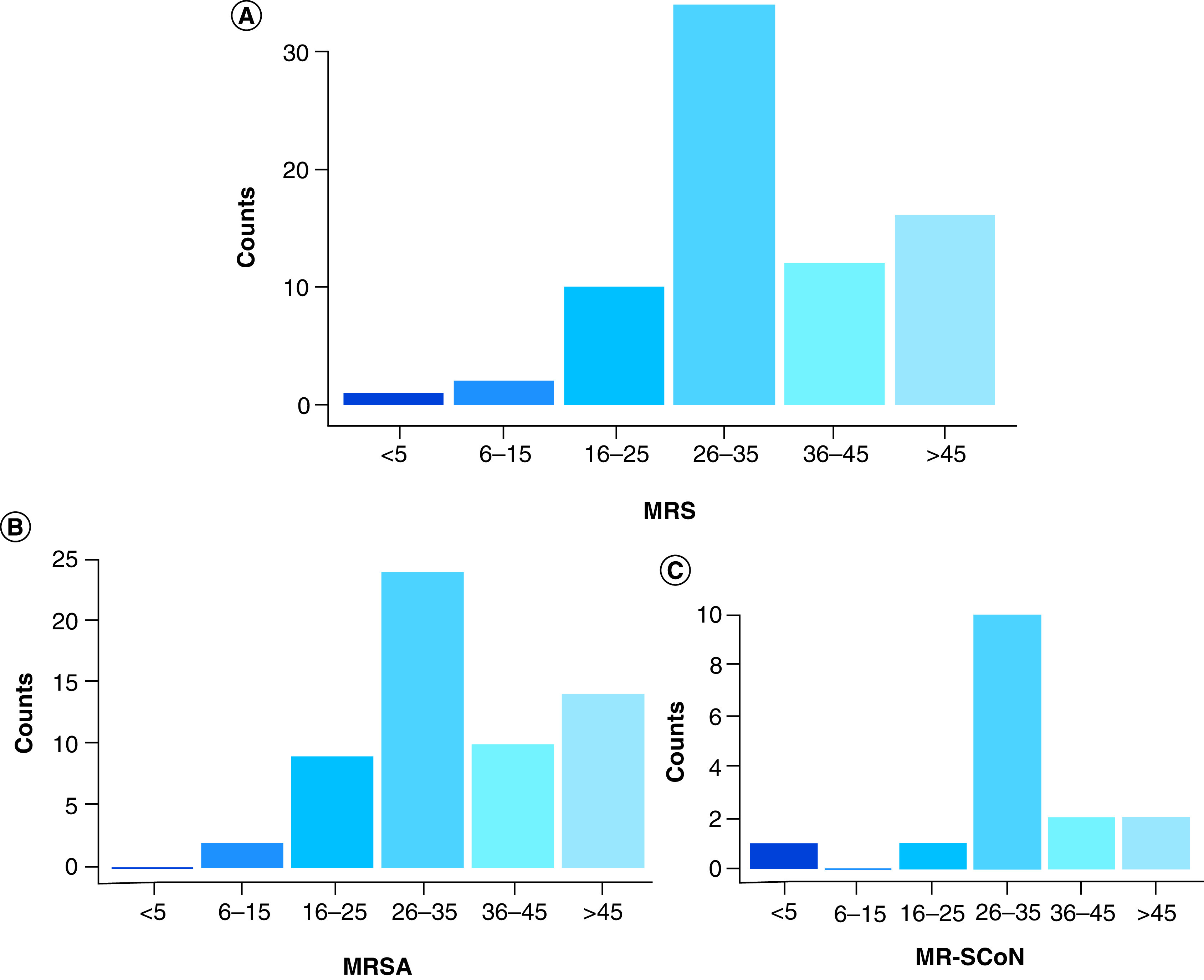
Distribution of methicillin-resistant staphylococci and methicillin-resistant staphylococci species per age group over a 3-year period. **(A)** Overall distribution of MRS per age group; **(B)** Distribution of MRSA per age group; **(C)** Distribution of MR-CoNS per age group. MR-CoNS: Methicillin-resistant coagulase negative staphylococci; MRS: Methicillin-resistant staphylococci; MRSA: Methicillin-resistant *Staphylococcus aureus*.

The mean age of patients infected by MRS was quite similar in female (35.17 years, SD ± 15.02) and males (34.70 years, SD ± 12.59) with 31 and 34 years being the median in females and males, respectively. The Chi-square analysis revealed that the number of patients with MRS infection increased significantly with age (χ^2^ = 16.936; p = 0.005). More specifically, the Figure 2 shows that MRS was more frequently detected in patients of ages within 26–35 years (34/75; 45.33%) and over 45 years (16/75; 21.33%). This age correlation was further observed to the species level where, MRSA prevalence was associated with similar age groups of 26–35 years (24/59; 40.68%) and over 45 years (14/59; 23.73%; Figure 2).

**Figure 3.**
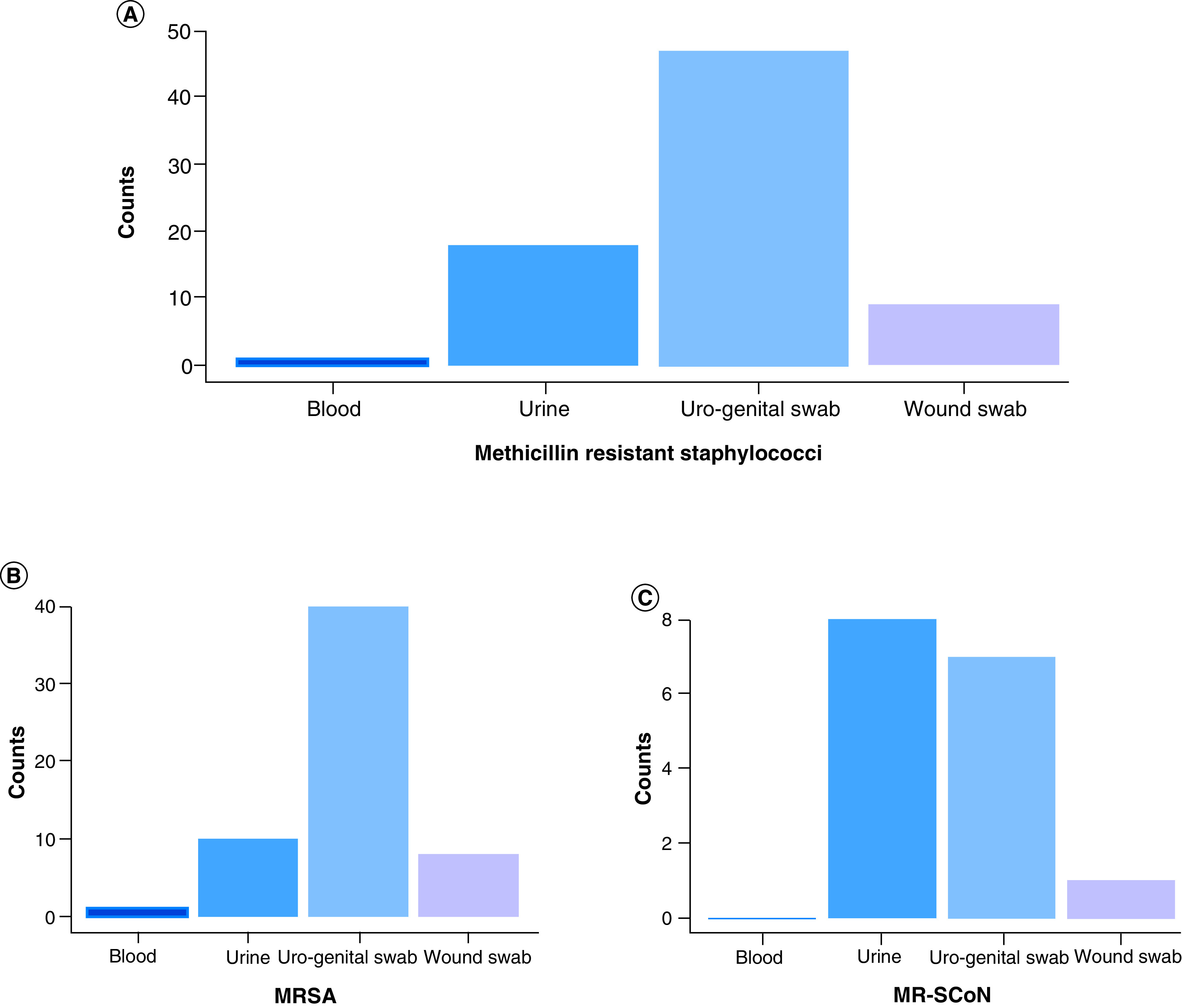
Distribution of methicillin resistant staphylococci and methicillin resistant staphylococci species per specimen type over a 3-year period. **(A)** Overall distribution of MRS per specimen type- **(B)** Distribution of MRSA per specimen type. **(C)** Distribution of MR-CoNS per specimen type. MR-CoNS: Methicillin resistant coagulase negative staphylococci; MRS: Methicillin resistant staphylococci; MRSA: Methicillin-resistant *Staphylococcus aureus*.

**Figure 4.**
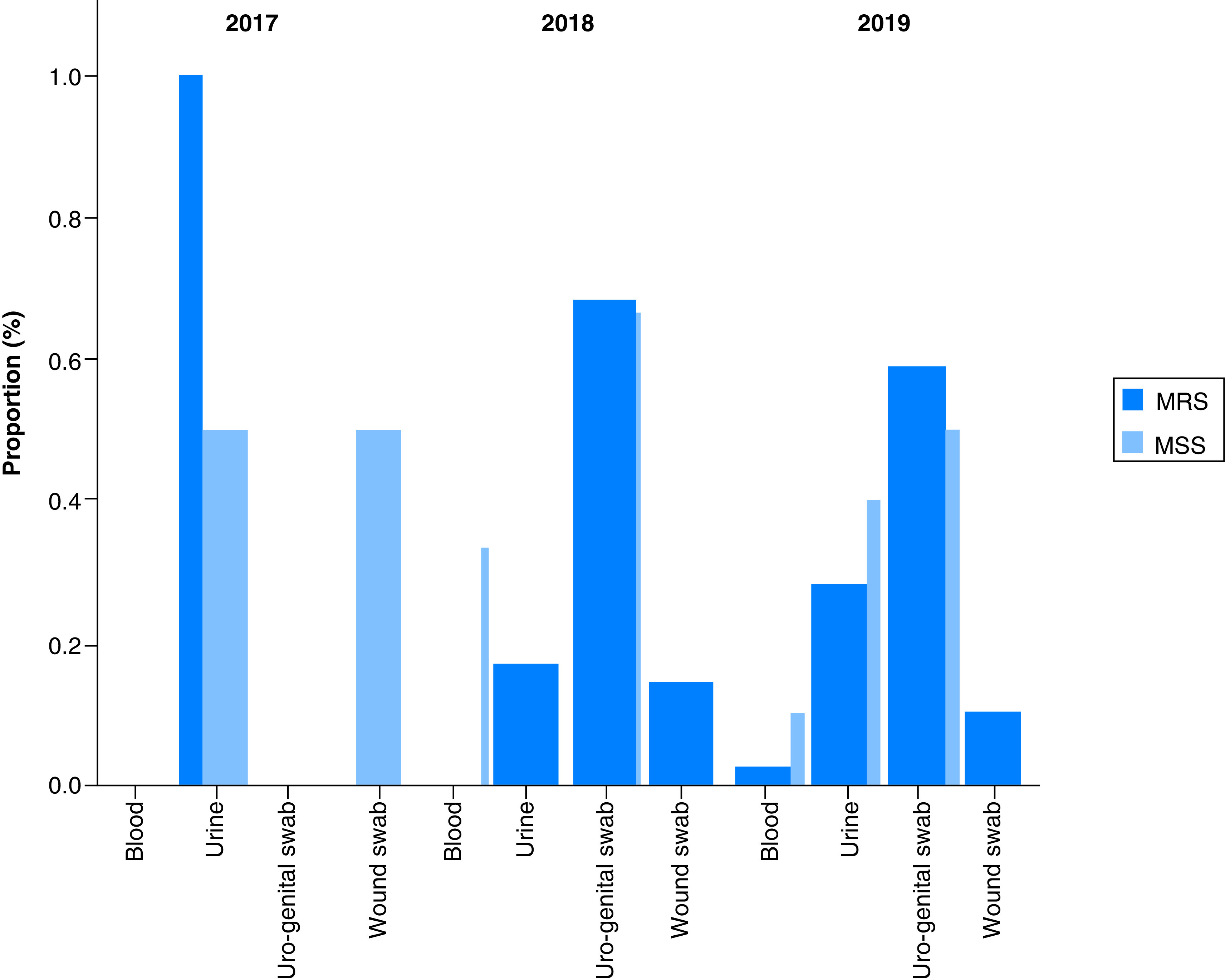
Overall distribution of methicillin-resistant staphylococci by specimen type and year. MRS: Methicillin-resistant staphylococci; MSS: Methicillin-susceptible staphylococci.

MRS were more prevalent in uro-genital swabs (62.67%, 47/75), followed by urine (24%, 18/75) and wound swabs (12%, 9/75) over the 3-year period (Figure 3). More specifically, in 2017, MRS were principally detected in urine whereas 68.6 and 58.97% of MRS were identified in uro-genital swabs in 2018 and 2019, respectively (Figure 4).

### Antimicrobial resistance patterns in MRS isolates

Overall, high level of resistance to penicillin (90%), cefoxitin (94%), trimethoprim-sulfamethoxazole (89%), tetracyclin (83%), doxycycline (84%), fosfomycine (71%) and ciprofloxacin (70%) was observed in MRSA isolates. Similarly, the most prevalent MR-CNS, *S. xylosus* displayed high resistance to cefoxitin (100%), netilmycin (100%), azithromycin (100%), tetracyclin (100%), fosfomycin (100%), trimethoprim-sulfamethoxazole (100%), ciprofloxacin (83%) and levofloxacin (75%). Methicillin resistant *S. lentus* and *S. epidermidis*, exhibited high resistance to penicillin (100%), cefoxitin (100%), tetracyclin (100%) and ciprofloxacin (100%) (Table 2).

**Table 2.** Distribution of methicillin resistant staphylococci isolated from clinical samples according to their species diversity and resistance level.

Bacterial species	P	FOX	CN	AK	Net	DA	AZI	E	CIP	LEV	TET	DOX	FF	FA	F	C	TMP/SXT
Over 3-year period																	
* S. aureus*	90%	94%	46%	24%	43%	35%	38%	64%	70%	59%	83%	84%	71%	44%	33%	37%	89%
* S. auricularis*	0%	0%	0%	0%	0%	0%	0%	0%	100%	0%	0%	0%	0%	0%	0%	0%	0%
* S. capitis*	0%	100%	0%	100%	0%	100%	0%	100%	0%	0%	100%	0%	0%	0%	0%	100%	0%
* S. epidermidis*	100%	100%	25%	0%	0%	33%	0%	67%	100%	100%	100%	0%	0%	0%	100%	50%	100%
* S. lentus*	100%	100%	25%	0%	0%	33%	0%	50%	100%	100%	100%	0%	0%	0%	0%	0%	0%
* S. warneri*	100%	100%	0%	0%	0%	100%	0%	100%	0%	0%	50%	100%	0%	50%	50%	100%	100%
* S. xylosus*	67%	100%	17%	0%	100%	0%	100%	40%	83%	75%	100%	67%	100%	0%	0%	50%	100%
2019																	
* S. aureus*	86%	89%	31%	27%	30%	31%	57%	66%	58%	53%	80%	85%	67%	60%	33%	50%	85%
* S. auricularis*	0%	0%	0%	0%	0%	0%	0%	0%	100%	0%	0%	0%	0%	0%	0%	0%	0%
* S. epidermidis*	100%	100%	0%	0%	0%	0%	0%	50%	100%	100%	100%	0%	0%	0%	100%	100%	0%
* S. lentus*	100%	100%	50%	0%	0%	0%	0%	0%	100%	100%	100%	0%	0%	0%	0%	0%	0%
* S. warneri*	100%	100%	0%	0%	0%	100%	0%	100%	0%	0%	0%	100%	0%	100%	100%	0%	0%
* S. xylosus*	0%	100%	0%	0%	0%	0%	100%	50%	100%	100%	100%	0%	100%	0%	0%	50%	100%
2018																	
* S. aureus*	100%	100%	75%	20%	75%	39%	17%	61%	83%	80%	100%	83%	75%	29%	33%	18%	100%
* S. capitis*	0%	100%	0%	100%	0%	100%	0%	100%	0%	0%	100%	0%	0%	0%	0%	100%	0%
* S. epidermidis*	100%	100%	100%	0%	0%	100%	0%	100%	0%	100%	0%	0%	0%	0%	100%	0%	100%
* S. lentus*	100%	100%	0%	0%	0%	100%	0%	100%	100%	100%	0%	100%	0%	0%	0%	0%	0%
* S. warneri*	100%	100%	0%	0%	0%	100%	0%	100%	0%	0%	100%	0%	0%	0%	0%	100%	100%
* S. xylosus*	100%	100%	50%	0%	100%	0%	100%	33%	67%	50%	100%	50%	0%	0%	0%	0%	100%
2017																	
* S. warneri*	0%	100%	0%	0%	0%	0%	0%	100%	0%	0%	0%	0%	0%	0%	0%	0%	0%
* S. xylosus*	0%	0%	0%	0%	0%	0%	0%	0%	0%	0%	100%	100%	0%	0%	0%	0%	0%

AK: Amikacin; AZI: Azithromycin; C:Chloramphenicol; CIP: Ciprofloxacin; CN: Gentamicin; DA: Clindamycine; DOX: Doxycyclin; E: Erythromycin; F: Nitrofurantoin; FA: Fusidic acid; FF: Fosfomycin; FOX: Cefoxitin; LEV: Levofloxacin; Net: Netilmicin; P: Penicillin; TET: Tetracyclin; TMP/SXT: Trimethoprim-Sulfamethoxazole.

Altogether, 65 resistance patterns were observed in MRS. Among these, 52 unique resistance patterns were detected in MRSA isolates with resistance to a minimum of two (n = 4) and a maximum of 12 antibiotics (n = 1). Around 70% of MRS were multi-drug resistant (MDR) with 69.49 and 68.75% of MRSA and MR-CoNS displaying MDR, respectively. The resistance profiles P.FOX.CIP, P.FOX.CIP.FF, FOX.DA.CIP and P.FOX.TE.DOX.TMP/SXT were more prevalent among MRSA (Table 3). The profile FOX.P.CN.VA.DA.E.LEV.TE.FF.FA.C.TMP/SXT, displayed concomitant resistance to 12 antibiotics belonging to ten antibiotic families followed by the resistance profile FOX.P.VA.DA.AMC.E.TE.DOX.FA.TMP/SXT showing resistance to ten antibiotics including last resort vancomycin. There were no predominant resistance patterns in MR-CoNS but the resistance profile FOX.P.CN.NET.E.CIP.LEV.SPA.TE.DOX exhibited resistance to ten antibiotics of six families of antibiotics, followed by the resistance profiles FOX.P.VA.DA.E.DOX.FA.F and FOX.P.CN.DA.E.LEV.F.TMP/SXT that were both resistant to eight antibiotics of six antibiotic families (Table 4).

**Table 3.** Antimicrobial resistance profiles of methicillin resistant *Staphylococcus aureus* isolated from clinical samples.

Resistance patterns (n = isolates)	Antibiotics (n)	Classes (n)
FOX.CIP.LEV.TE (1)	4	3
FOX.CN (1)	2	2
FOX.CN.DA.E.SPA.DOX.FA.TMP/SXT (1)	8	7
FOX.DA.CIP (2)	3	3
FOX.DA.E.CIP (1)	4	3
FOX.DA.E.CIP.LEV (1)	5	3
FOX.DOX (1)	2	2
FOX.E.SXT (1)	3	3
FOX.NET.VA.E.TMP/SXT (1)	5	5
FOX.P (1)	2	1
FOX.P.AK.NET.TE.DOX (1)	6	4
FOX.P.AMC.TMP/SXT (1)	4	2
FOX.P.AZI.CIP (1)	4	3
FOX.P.AZI.CIP.FA (1)	5	4
FOX.P.C.TE.E (1)	5	4
FOX.P.CIP.FF (2)	4	3
FOX.P.CN (1)	3	2
FOX.P.CN.AK (1)	4	2
FOX.P.CN.AK.E.TE.DOX.FF.FA.C (1)	10	8
FOX.P.CN.AK.FA.TMP/SXT.CIP (1)	7	5
FOX.P.CN.AZI.TE.C (1)	6	5
FOX.P.CN.E.CIP.TE.C. (1)	7	6
FOX.P.CN.NET.CIP.LEV.TE.DOX (1)	8	5
FOX.P.CN.NET.DA.E.AZI (1)	7	3
FOX.P.CN.NET.E.AZI.FA (1)	7	4
FOX.P.CN.NET.VA.DA.E.LI.FA.C (1)	10	6
FOX.P.CN.VA.DA.E.CIP.DOX (1)	8	6
FOX.P.CN.VA.DA.E.CIP.DOX.FA (1)	9	7
FOX.P.CN.VA.DA.E.DOX.C (1)	8	6
FOX.P.CN.VA.DA.E.LEV.TE.FF.FA.C.TMP/SXT (1)	12	10
FOX.P.CN.VA.DA.E.TE.F.C (1)	9	7
FOX.P.DA.E.LEV.TE (1)	6	4
FOX.P.DA.E.LEV.TMP/SXT (1)	6	4
FOX.P.DA.E.TE.FF.FA (1)	7	5
FOX.P.E (1)	3	2
FOX.P.E.TE.FA.TMP/SXT (1)	6	5
FOX.P.E.TE.TMP/SXT (1)	5	4
FOX.P.FA (1)	3	2
FOX.P.FA.FF.CIP (1)	5	4
FOX.P.FF.F (1)	4	3
FOX.P.LEV.DOX.FA (1)	5	4
FOX.P.NOR.LEV (1)	4	2
FOX.P.TE.DOX.TMP/SXT (2)	5	4
FOX.P.TMP/SXT.FF (1)	4	3
FOX.P.VA.DA.AMC.E.TE.DOX.FA.TMP/SXT (1)	10	7
FOX.TMP/SXT (1)	2	2
FOX.VA.DA.E.DOX.FA (1)	6	5
FOX.VA.TMP/SXT (1)	3	3
P.FOX.CIP (2)	3	2
P.FOX.CIP.FA (1)	4	3
P.FOX.CIP.FF.TMP/SXT (1)	5	4
P.FOX.CIP.LEV (1)	4	2

AK: Amikacin; AZI: Azithromycin; C: Chloramphenicol; CIP: Ciprofloxacin; CN: Gentamicin; DA: Clindamycine; DOX: Doxycyclin; E: Erythromycin; F: Nitrofurantoin; FA: Fusidic acid; FF: Fosfomycin; FOX: Cefoxitin; LEV: Levofloxacin; Net: Netilmicin; P: Penicillin; TET: Tetracyclin; TMP/SXT: Trimethoprim-sulfamethoxazole.

**Table 4.** Antimicrobial resistance profiles of methicillin resistant coagulase negative staphylococci isolated from clinical samples.

*Staphylococcus* spp. (n = isolates)	Resistance patterns (n = isolates)	Antibiotics (n)	Classes (n)
*S. xylosus* (5)	FOX.AZI.CIP.TE.C.TMP/SXT (1)	6	6
	FOX.CIP.LEV.FF.VA (1)	5	4
	FOX.NAL (1)	2	2
	FOX.P.AZI.CIP.TMP/SXT (1)	5	4
	FOX.P.CN.NET.E.CIP.LEV.SPA.TE.DOX (1)	10	6
*S. lentus* (4)	FOX.DA.E.NET.DOX (2)	5	4
	FOX.P.CN.NOR.CIP (1)	5	3
	FOX.P.E (1)	3	2
*S. warneri* (3)	FOX.E (1)	2	2
	FOX.P.DA.E.TE.C.TMP/SXT (1)	7	5
	FOX.P.VA.DA.E.DOX.FA.F (1)	8	6
*S. epidermidis* (4)	FOX.P (1)	2	1
	FOX.P.CN.DA.E.LEV.F.TMP/SXT (1)	8	6
	FOX.P.E.CIP.F.C.TE (1)	9	6
	P.FOX.CIP.LEV (1)	4	2
*S. capitis* (1)	FOX.AK.VA.DA.E.TE.C (1)	7	6

AK: Amikacin; AZI: Azithromycin; C:Chloramphenicol; CIP: Ciprofloxacin; CN: Gentamicin; DA: Clindamycine; DOX: Doxycyclin; E: Erythromycin; F: Nitrofurantoin; FA: Fusidic acid; FF: Fosfomycin; FOX: Cefoxitin; LEV: Levofloxacin; Net: Netilmicin; P: Penicillin; TET: Tetracyclin; TMP/SXT: Trimethoprim-Sulfamethoxazole.

## Discussion

MRSA was recognized as pathogen of high priority for research and development of new antibiotics by the WHO [7] given its serious socio-economic repercussions globally. In this study, the prevalence and antimicrobial resistance patterns of MRS isolated from clinical samples in a private laboratory in Cameroon were retrospectively analyzed.

Of the 1683 samples analyzed over the 3-year period, 90 (5%) staphylococcal isolates were recorded. The results obtained in the present study demonstrated the high prevalence of MRS (83%) with 78.67% being MRSA. The MRS prevalence reported in our study is consistent with studies from Cameroon where 72 and 75% of MRS were detected in clinical [3] and carriage [1] samples in Yaounde, respectively. In contrast, the results are lower than that reported from a survey conducted in a tertiary hospital in Northern Thailand where a maximum prevalence of MRS (100%) was detected from clinical isolates [8]. These results are however, higher in comparison with that reported in studies from other part of the world. A report from Congo demonstrated a 63.5% prevalence of MRS among hospitalized surgical patients with 63.5 and 60% being MRSA and MR-CNS, respectively [9]. Likewise, a 4-year cross-sectional study conducted in India revealed a 34% prevalence of MRS from clinical specimens [10], while Ramsamy *et al.* showed a decreasing MRSA prevalence ranging from 28% to 18% over a 5-year period (2011–2015) in KwaZulu-Natal, South Africa [11]. In contrast to low-and-middle-income countries, lower MRSA prevalence (<25%) has been reported in European countries [2].

These discrepancies could be explained by the lack of effective implementation of infection, prevention and control measures in hospitals as well as irrational use of antibiotics in resource-constrained settings [4]. Moreover, the high MRS prevalence, especially MRSA, observed in our study could be attributed to the high burden of infectious diseases coupled with suboptimal hygiene and sanitation. Additionally, given that antimicrobial resistance is a neglected concern and antibiotic use is not necessarily well regulated in Cameroon, lack of monitoring, prevention and control measures likely engender extensive antibiotic consumption and subsequent high resistance rates in the country. Comprehensively delineate the molecular nature and epidemiology of MRS circulating in communities and hospitals in Cameroon is required in order to inform evidence-based strategies for rapid monitoring, prevention and containment of antimicrobial resistance.

Our findings revealed that the prevalence of MRS varied across year. Although, these changes may be attributable to shifts in incidence between hospital and community-based patients, we postulated that the variation observed could also be related to several factors including difference in demographics (ethnicity, socio-economic status, geography, etc.), practice variability among clinicians and laboratorians, as well as seasonal variation. This suggests that population-based studies are required to establish appropriate distribution and determinants of MRS infectious diseases.

Overall, females were more positive to MRS than males. It is unclear, why females were more positive than males especially given that previous reports revealed that males are more prone to bacterial carriage and infection albeit females might have a poorer outcome [12,13]. Our result shows that further research investigating the relationship between gender and risk of infection, the reason for higher MRS infection rates in females as well as other factors of infection incidence such as age and comorbid conditions are required.

The analyses of resistance profiles revealed that MRS isolates exhibited high level of MDR (70%) with >90% resistance to β-lactams including penicillin and cefoxitin for both MRSA and MR-CNS. This MDR prevalence is lower than that reported in a previous Cameroonian study where 100% of MRSA were MDR [3]. Co-resistance to non-β-lactam antibiotics, including trimethoprim-sulfamethoxazole, ciprofloxacin, tetracyclin, doxycyclin, gentamicin and fosfomycin was also observed. Such co-resistance to β-lactams together with other antibiotic families is frequent in MRS and could likely result from the indiscriminate and/or extensive antibiotic use (misuse, overuse and inappropriate use) in the country [14]. The co-resistance could also be explicated by the presence of mobile genetic elements such as plasmids, integrons, transposons and insertion sequences that are responsible of further resistance. One MRSA isolate displayed the phenotype FOX.P.CN.VA.DA.E.LEV.TE.FF.FA.C.TMP/SXT showing resistance to 12 antibiotics including the last resort drug vancomycin while one MR-CONS isolate displayed the profile FOX.P.CN.NET.E.CIP.LEV.SPA.TE.DOX exhibited resistance to ten antibiotics.

It is noteworthy to mention that the heterogeneity of the samples that were collected from both clinically ill and asymptomatic patients preclude any conclusion it was not possible to distinguish between infection from colonization.

## Conclusion

The high prevalence of MRS and MDR MRSA and MR-CONS observed in this study highlights the need to strengthen antimicrobial stewardship and infection, prevention and control programs in the country. More multidimensional molecular epidemiological studies are urgently needed if the country is to reach United Nations Sustainable Development Goal (UN SDG) three of ensuring healthy lives and promoting well-being for all.

## Limitations

Heterogeneity of the samples preclude robust conclusion regarding the association of MRS with infections given that sample were collected from both clinically ill and apparently asymptomatic patients. Moreover, although being performed over a 3-year period, the study was conducted in a single geographic area, hence the findings might not represent the broad population and whole country. The absence of molecular tests and genotyping also hinder appropriate molecular epidemiological data that could be used not only to prevent outbreak situations but also to invigorate adequate antibiotic resistance containment measures.

## Future perspective

This study underscores the importance of routine screening, and monitoring of MRS as with other resistant bacteria. It further reveals that molecular techniques despite being uneconomical at small scale should also be integrated in the diagnostic confirmation of infectious diseases and especially MDR in developing countries such as in Cameroon. Upcoming efforts for better understanding of MRS and MRSA should therefore focus on two main areas namely: host and pathogen interaction and evaluation of MRS genomics, proteomics, metabolomics and epigenetics in animal models. More imminently, high-quality clinical and molecular epidemiological studies are needed to inform appropriate public health strategies and interventions. The study revealed that females were more infected by MRS than males, thus future studies investigating MRS in neonates should further be implemented to evaluate the incidence of MRS on neonates born from a similar cohort.

**Summary points**

- Methicillin-resistant staphylococci (MRS) are major source of infections in hospital and community settings.
- Trends and antimicrobial resistance profiles of circulating MRS strains were ascertained in Yaoundé, Cameroon.
- MRS was detected in over 80% of positive specimen with 79% being methicillin-resistant *Staphylococcus aureus* (MRSA).
- Altogether, 65 resistance patterns were observed in MRS with resistance to a minimum of two (n = 4) and a maximum of 12 antibiotics (n = 1).
- MRS isolates displayed around 70% of multi-drug resistance with both MRSA and methicillin-resistant coagulase-negative staphylococci displaying similar multi-drug resistance prevalence (around 70%).
- The prevalence of MRS infection increased significantly with age.
- The high prevalence of MRS especially of MRSA observed in this study highlights the need to strengthen antimicrobial stewardship and infection, prevention and control programs in the country.
- Our findings reinforce the need to strengthen comprehensive multidimensional molecular epidemiological studies if the country is to reach United Nations Sustainable Development Goal (UN SDG) three of ensuring healthy lives and promoting wellbeing for all.
